# Oncologists' Communication About Uncertain Information in Second Opinion Consultations: A Focused Qualitative Analysis

**DOI:** 10.3389/fpsyg.2021.635422

**Published:** 2021-05-31

**Authors:** Jamie L. van Someren, Vicky Lehmann, Jacqueline M. Stouthard, Anne M. Stiggelbout, Ellen M. A. Smets, Marij A. Hillen

**Affiliations:** ^1^Department of Medical Psychology, Amsterdam Public Health, Amsterdam University Medical Centers, University of Amsterdam, Amsterdam, Netherlands; ^2^Department of Medical Oncology, Netherlands Cancer Institute, Amsterdam, Netherlands; ^3^Medical Decision Making, Department of Biomedical Data Sciences, Leiden University Medical Center, Leiden, Netherlands

**Keywords:** communication, second opinion, oncology, uncertainty, physician-patient relations, patient-centered communication

## Abstract

**Introduction:** Uncertainty is omnipresent in cancer care, including the ambiguity of diagnostic tests, efficacy and side effects of treatments, and/or patients' long-term prognosis. During second opinion consultations, uncertainty may be particularly tangible: doubts and uncertainty may drive patients to seek more information and request a second opinion, whereas the second opinion in turn may also affect patients' level of uncertainty. Providers are tasked to clearly discuss all of these uncertainties with patients who may feel overwhelmed by it. The aim of this study was to explore how oncologists communicate about uncertainty during second opinion consultations in medical oncology.

**Methods:** We performed a secondary qualitative analysis of audio-recorded consultations collected in a prospective study among cancer patients (*N* = 69) who sought a second opinion in medical oncology. We purposively selected 12 audio-recorded second opinion consultations. Any communication about uncertainty by the oncologist was double coded by two researchers and an inductive analytic approach was chosen to allow for novel insights to arise.

**Results:** Seven approaches in which oncologists conveyed or addressed uncertainty were identified: (1) specifying the degree of uncertainty, (2) explaining reasons of uncertainty, (3) providing personalized estimates of uncertainty to patients, (4) downplaying or magnifying uncertainty, (5) reducing or counterbalancing uncertainty, and (6) providing support to facilitate patients in coping with uncertainty. Moreover, oncologists varied in their (7) choice of words/language to convey uncertainty (i.e., “I” vs. “we”; level of explicitness).

**Discussion:** This study identified various approaches of how oncologists communicated uncertain issues during second opinion consultations. These different approaches could affect patients' perception of uncertainty, emotions provoked by it, and possibly even patients' behavior. For example, by minimizing uncertainty, oncologists may (un)consciously steer patients toward specific medical decisions). Future research is needed to examine how these different ways of communicating about uncertainty affect patients. This could also facilitate a discussion about the desirability of certain communication strategies. Eventually, practical and evidence-based guidance needs to be developed for clinicians to optimally inform patients about uncertain issues and support patients in dealing with these.

## Introduction

Cancer treatment has become increasingly complex, involving various treatment modalities that affect tumor growth and side effects in a multitude of ways, making it difficult to predict outcomes/prognosis for individual patients. The meaning and implication of diagnostic tests may also be ambiguous, further adding to high levels of uncertainty in oncology (Parascandola et al., 2002; Politi et al., 2007; Han et al., 2011, 2019; Politi and Street, 2011; Simpkin and Armstrong, 2019). For oncologists it can be complex and demanding to discuss these various uncertainties. Yet, fully informing patients and involving them in medical decision-making is becoming the norm in healthcare and is considered to be a physician's ethical duty (Han et al., 2011, 2019; Balogh et al., 2015; Bhise et al., 2018; Blanch-Hartigan et al., 2019; Simpkin and Armstrong, 2019). Moreover, managing uncertainty is considered one of the key components of patient-centered communication (Epstein and Street, 2007). When consulting with cancer patients who seek a second opinion (SO), providers need to deal with additional uncertainties, given potential discrepancies with the first opinion and/or potential new treatment options. Thus, discussing uncertainty in the setting of oncological SO consultations can be particularly challenging.

Cancer patients themselves have indicated wanting to be fully informed about diagnosis, prognosis, treatment, and side effects, even if the information contains uncertainties (Blanchard et al., 1988; Quill, 2000; Jenkins et al., 2001; Cox et al., 2006; Murtagh and Thorns, 2006; Hancock et al., 2007; Parker et al., 2007; Evans et al., 2009; Ahalt et al., 2012). At the same time, some patients may feel burdened and emotionally overwhelmed by uncertainty (Arora, 2003; Politi et al., 2007; Han, 2013). For example, patients have been found to interpret uncertain information (e.g., risk estimates) too pessimistically (Han, 2013). Awareness of uncertainty may also increase their cancer-related worries and fears, and may rush them into rapid treatment initiation (Denberg et al., 2006; Mishel et al., 2009; Han et al., 2011). Moreover, uncertainty may be an important motivator for cancer patients to seek a second opinion (SO), in an effort to reduce uncertainty (Kurian et al., 2017; Shmueli et al., 2017; Blanch-Hartigan et al., 2019). An oncological SO may indeed reduce uncertainty, for example if it confirms the first opinion. In contrast, a SO may increase uncertainty if it yields additional or even contradicting new information/options (Hillen et al., 2017a,c).

How oncologists discuss uncertain information may be crucial for patients' ability to cope with uncertainty, as indicated in previous research. For example, both in and outside the oncology setting, patients were less trusting of physicians and less satisfied if they expressed uncertainty, as it reduced patients' perceived competence of the physician (Parascandola et al., 2002; Blanch et al., 2009; Cousin et al., 2013). In contrast, other studies reported improved patient satisfaction if physicians expressed uncertainty (Gordon et al., 2000). These contradictory effects may partially be explained by the finding that physicians who expressed more uncertainty also used more positive talk, relationship building, and provided more information to patients (Gordon et al., 2000; Blanch-Hartigan et al., 2019). In other words, *how* uncertain information is communicated may affect patients' perceptions, decisions, and ultimately their well-being.

Practical advice for clinicians on how to communicate uncertainty has been put forth, but empirical evidence to substantiate it is lacking (Han et al., 2019; Simpkin and Armstrong, 2019). Moreover, there is currently limited observational evidence on how healthcare providers communicate about uncertain issues with patients, particularly in highly uncertain settings. Therefore, this study aimed to provide an overview of approaches that oncologists use to discuss uncertain information during SO consultations in medical oncology, which are characterized by high levels of uncertainty. This overview will enable future research to assess the effects of different communication approaches on patients, and develop evidence-based recommendations for communicating uncertain information with patients.

## Materials and Methods

We present a secondary analysis of data collected in a prospective longitudinal study on communication during SO consultations, the SO-COM study (Lehmann et al., 2020). Data were collected between 2018 and 2019 and the larger study included self-report and observed behavioral data coding of audio-recorded SO consultations. Medical oncologists at two Dutch tertiary referral centers were invited to participate and signed informed consent forms. Patients (treated anywhere in the Netherlands) who were scheduled for a SO with participating oncologists were contacted by the hospitals to introduce the SO-COM study. Interested patients were subsequently called by the research team, and after verbally consenting they were sent informed consent forms and information to complete surveys. SO consultations were audio-recorded by dedicated research staff (not present during the SO). Confidentiality was guaranteed at all times and all procedures were approved by our local ethical committee (NL63087.018.17).

### Sample Selection

Eligible participants for the SO-COM study were adult cancer patients with any type of solid tumor, and who were proficient in the Dutch language (Lehmann et al., 2020). A total of *N* = 69 SOs were audio-recorded and for the current qualitative analysis, a purposive selection of audio-recorded consultations was used.

To create maximum variation in communication about uncertainty, we deliberately selected SO consultations based on two characteristics expected to be strongly associated with such communication: (1) the degree of patient-centered communication (PCC) by the oncologists and (2) oncologists' gender. First, PCC can be defined as physician behaviors which enable patients to express their perspectives on illness, treatment and health-related behavior, including symptoms, concerns and expectations (page 662; Zandbelt et al., 2005). Because “uncertainty management” is a key component of PCC (Epstein and Street, 2007), we hypothesized that oncologists' use of PCC would be associated with their communication about uncertainty. Therefore, we purposively selected the *n* = 6 highest and *n* = 6 lowest PCC-scoring consultations for qualitative analysis (*N* = 12). As part of the larger SO-COM study, PCC scores had been rated by trained coders, based on three items of the Euro-communication scale (Mead and Bower, 2000), focusing on whether the oncologists encouraged patients to express themselves, listened, and involved them in any decisions. Second, previous findings suggest that physician's sex may determine how they communicate uncertainty. For example, females may convey uncertainty more apologetically than males (Schumann and Ross, 2010) and female physicians used more non-verbal indicators of uncertainty than male physicians (Blanch et al., 2009). Therefore, we expected that purposive selection for physician sex would enhance variability in our data. We ensured equal representation of both sexes (*n* = 6 each), and selected the *n* = 3 lowest scoring (on PCC) SOs by male and *n* = 3 lowest scoring by female oncologists, and did the same for the highest scoring consultations (i.e., *n* = 3 male, *n* = 3 female). We further increased variability by selecting only one consultation per oncologist (i.e., 12 out of 24 different oncologists were included; see Results section). We closely monitored whether data saturation was achieved after analysis of our initial selection of 12 consultations. We concluded this was the case, as indicated by the two final consultations not yielding any significant new information (Francis et al., 2010).

### Qualitative Data Analysis

The 12 purposively selected audio-recorded SOs were transcribed verbatim. Any consultation segments involving talk about the patient's medical history, personal life, or scheduled follow-up appointments, as well as small talk about non-medical issues (e.g., the weather) were first checked. If they did not contain any talk about uncertainty by the consulting oncologist (e.g., treatment options, side-effects, risks, recurrence), these segments were not transcribed and excluded from the analysis. All coding was performed using MAXQDA 2020 (VERBI Software, 2019). An inductive constant comparative approach was chosen to ensure that analysis was data-driven rather than informed by existing literature or a theoretical framework (Strauss, 1987; Boeije, 2002). We coded both verbal expressions of uncertainty that were made by the oncologist spontaneously, as well as those in response to the patient's expression of uncertainty. Communication about uncertainty was defined according to Han et al.'s (2011) definition of uncertainty as any talk by the oncologist indicating his/her subjective awareness of ignorance (p.830). Hence, this included any uncertainty about the likelihood of a future event (e.g., risks of side-effects), uncertainty due to limited available information (e.g., inconsistent test results) or due to complexity (e.g., the interplay between a multitude of causal factors) (Han et al., 2011). All 12 SOs were double-coded by two coders independently (JLS and MH) and subsequently discussed together with VL to resolve issues and constantly adjust the coding scheme. This procedure of researcher triangulation was employed to ensure that multiple perspectives and possible interpretations of the data were incorporated. All codes were clustered into overarching themes using thematic analysis through continuous discussions within the research team. After initial themes were identified, potentially disconfirming evidence was sought in our data and not identified, thus further enhancing the validity of our results (Creswell and Miller, 2000).

## Results

### Patient Sample

Cancer patients in the 12 SO consultations were on average 53 years old (range 28-85), *n* = 7 were female (58.3%) and *n* = 5 male (43.7%). They had varying educational backgrounds, including high-level education (i.e., college/university, *n* = 5; 41.7%), middle (i.e., secondary vocational training, *n* = 4; 33.3%), and lower education (i.e., high school or low vocational training, *n* = 3; 25%). The majority of patients were in an advanced stage of their disease (*n* = 10; 83.3%) and the most prevalent diagnoses were breast cancer (*n* = 4; 33.3%) and gastrointestinal tumors (*n* = 4; 33.3%). Duration of the selected SO consultations ranged between 27 and 61 min (*M* = 41 min).

### Communication About Uncertainty

From the qualitative data analysis, seven different approaches to communicating with patients about uncertainty emerged (see Supplementary Table 1): (1) specifying the degree of uncertainty, (2) explaining reasons of uncertainty, and (3) providing personalized estimates of uncertainty to patients. Moreover, it appeared that oncologists pursued certain goals by (4) downplaying or magnifying uncertainty, (5) reducing or counterbalancing uncertainty, or (6) providing support to facilitate patients in coping with uncertainty. Finally, we found variation in oncologists' (7) choice of words/language to convey uncertainty. Although these approaches are presented separately, some may directly follow each other, while specific overlap between them was present in the consultations. For example, specific use of language to express uncertainty (strategy 7) co-occurred with all other strategies.

Discussions of uncertainty were either initiated by the oncologist or in response to patients' expressions of uncertainty, such as questions about life expectancy. Discussions of uncertainty were not limited to specific parts of the consultations, but were present throughout all phases of the consultations. Moreover, we did not identify a consistent “style” of discussing uncertainty by individual oncologists: oncologists varied widely in their use of the identified approaches, both between and within consultations. For example, within one consultation an oncologist could very explicitly express uncertainty about one topic, yet implicitly discuss another uncertain topic.

#### Specifying the Degree of Uncertainty

Oncologists varied in the degree of specifying uncertain information, particularly when talking about prognostic matters, such as the risk of side effects or potential success rates of certain treatments. On one end of the spectrum, they would remain rather vague, by using generic words (e.g., *rather, much, many patients*) to describe how uncertain a situation or risk was. In contrast, oncologists would occasionally provide additional quantification, for example by providing qualitative utterances along with specific estimates. For example, “*The [chemotherapy] works for approximately 20%, that is one in five patients, and we still can't predict precisely for whom it will work.”* (male oncologist, male patient)

We did not identify certain patterns among oncologists' use of either strategy. However, overall it appeared that oncologists refrained from specifying the degree of uncertainty and remained rather vague in case of highly unpredictable outcomes, such as in the following example.

*Everything changed after the tumor responded well [to therapy]. Thus, the chance of recurrence at the start [of treatment] is very different from the chance after having had surgery. That is something to keep in mind: the chance [of recurrence] became a lot smaller*. (male oncologist, female patient).

#### Explaining Reasons of Uncertainty

Oncologists would sometimes explain underlying causes that made a situation uncertain or explain why they could not provide more precise estimates. Thereby, oncologists would explain the boundaries of medical testing, therapies, or science in general to emphasize that some uncertainty was unavoidable and omnipresent in cancer care.

*We never know in advance whether a cancer cell is left behind somewhere outside the surgical area. You can't see that, you only know once the disease recurs and realize that you weren't able to remove everything, because something started growing again. We have no method, no test to measure that beforehand*. (female oncologist, female patient)

In other situations, oncologists openly admitted and attributed their uncertainty to the limits of their own personal expertise.

***Patient:*** *What do you think of their [other hospital's] advice to radiate 15 times?*

***Oncologist:*** *Well, I'm not a radiologist, so I should stay within my own field of expertise. […] But I will discuss it with the radiologist in this hospital, because I don't think they would give that much radiation, but I'm not sure*. (female oncologist, female patient).

#### Providing Personalized Estimates of Uncertainty to Patients

Oncologists would sometimes provide a personalized estimate of uncertain information based on patients' individual characteristics, even when the evidence was scarce (first example below). Communicating tailored information may increase patients' understanding about their own disease and treatment trajectory. Oncologists appeared to use such strategies in an effort to reduce patients' feelings of uncertainty (second example below).

*In your case, where the disease returned in your abdomen after surgery, we don't know how much added value [another] surgery would have over this [other treatment]. Based on data from the past, we still think it would improve your chances somewhat. (2018)*

*We could give you only the first line of chemotherapy. […] Or we could consider to give you the second line of treatment simultaneously […]. Reasons to consider that option are as follows: the [metastases] are growing pretty fast, secondly: you're young, you're fit, and yes I think you could handle it. […] So that could be an option which you have to think about yourself, because it does mean that you will have more side effects from the treatment. But it also means that you will get a more powerful treatment all at once*. (female oncologist, male patient).

#### Downplaying or Magnifying Uncertainty

In some instances, oncologists appeared to purposively downplay or magnify uncertain information. In doing so, they seemingly attempted to persuade patients, steer their perceptions, or possibly even influence their behavior in a certain direction. For example, if oncologists clearly had a certain treatment preference, they would magnify uncertainty regarding options they did not prefer and/or downplay uncertainty related to their preferred option. The following example illustrates a case where the oncologist is transparent about her treatment preference and only highlights the positive side of this option, while ignoring possible drawbacks and thereby downplaying the risk of side effects.

*I would encourage you to choose this treatment. It's very different from what you had before. In general, it's well-tolerated. People work with it, do their daily activities. You won't experience hair loss. So that's great. You won't feel nauseous, people travel around the world with it really*. (female oncologist, female patient)

In other cases, oncologists would magnify uncertainty about what it would mean to participate in a clinical trial, and did not mention any potential advantages. They appeared to do so in an effort to steer the patient away from this option (first example below), and/or to temper patients' (unrealistic) hope (second example below).

*[The trial is] basically a lottery, so half [of the patients] get a pill with nothing in it and the other half gets [medication name]. But because it's a lottery, there's a 50% chance that you get nothing. So I think that's a disadvantage in itself. And the second [disadvantage] is that we don't really know if [the medication] is as good as the other treatment. Whereas, we do know about that [other] treatment that it cuts your risk for a relapse in half*. (female oncologist, female patient)

*I think it's complex. I certainly want to brainstorm with you, but I don't want to create false expectations. When I consider this trial, you may actually be too fragile to participate. In such a trial we give medication to patients, that have not been given to people before*. (male oncologist, male patient)

Due to our study setting in SO consultations, uncertainty about whether or not it was possible for patients to switch hospitals was present occasionally. Oncologists would sometimes magnify uncertain factors associated with a treatment transfer (e.g., waiting times) in an apparent effort to discourage patients from pursuing this option.

*If you say “I don't care, I still want that treatment here,” then of course I will consult with my surgeons about when I could get it done here. However, an important factor is also the waiting time, to be honest. In the end, if I were you, I would choose to have surgery in the place with the first availability. You said they could do it July 19th already? I'm afraid it would be August here*. (female oncologist, female patient).

#### Reducing or Counterbalancing Uncertainty

In response to patients' spontaneous questions or expressions of uncertainty, oncologists would sometimes react by trying to reduce the emotional burden of uncertainty. For example, they would directly provide information in an effort to reassure patients of certain aspects.

***Patient:*** *Doesn't the risk [of recurrence] increase if I stop this [hormone] therapy, or can it lead to a reversed effect?*

***Oncologist:*** *That the 3 years [of hormonal therapy] will [backfire]? No. Actually, the 3 years [of therapy that you had] are in your pocket, and no one can take that from you*. (female oncologist, female patient)

Alternatively, oncologists would offer specific ideas and explain actions that could be taken to actively reduce patients' uncertainty. For example, the oncologist below proposes a plan to reduce uncertainty that the patient expressed about the origin of his fatigue as a side effect of his current treatment.

*[…] My advice would be to stop [current treatment]. See what happens to your energy levels. Make a new scan after 2 months, and if something turns out to be active, you have two options: either try out this treatment, or in the most extreme case you could start that other treatment*. (male oncologist, male patient)

In other instances, oncologists would introduce uncertainty themselves, but counterbalance it right away by emphasizing aspects that *were* certain. Such counterbalancing appeared to be done in an effort to minimize the psychological burden of uncertainty on patients.

*It may still be possible that no cells traveled from the left [breast] to other parts of the body, that is possible. It is also possible that they did, but that those cells cannot grow. We don't know, and we don't have good tests to find that out. What we do know is that in large groups of women, who had this follow-up treatment, the chance of recurrence is two times smaller than for those without this follow-up treatment*. (female oncologist, female patient).

#### Providing Support to Facilitate Patients in Coping With Uncertainty

Oncologists occasionally explicitly tried to support patients emotionally in coping with uncertainty that patients either brought up during the consultation or had beforehand. One way of offering support was through directly asking about or addressing patients' and/or their relatives' worries and emotional reactions to uncertainty.

*I've only known you for a bit, but I'm trying to discover why you're anxious, and at the same time to help you. What are you worried about? I can imagine about a lot, but I wanted to ask it as an open question*. (male oncologist, wife of male patient)

In other instances, oncologists normalized worries and uncertainty by comparing an individual patient's situation with other patients and/or by placing it into a broader picture.

***Patient:*** *Are there other women who, like me, have stopped or want to stop [adjuvant hormonal treatment]? And how are they doing after a couple of years?*

***Oncologist:*** *Yes, many. That is always good to remember: you're definitely not the only one. I think that 40% of women don't complete the 5 years [of treatment]. Simply because it can cause some pretty bad side effects. And it's certainly true that the moment you use it less [frequently than intended], you have less effect, and then the cancer can recur. But there are also lots of these women for whom the cancer does not recur*. (male oncologist, female patient)

Moreover, oncologists provided support by discussing different scenarios that could be useful to the patient in the future. In the following example, the oncologist tries to offer a roadmap to help the patient cope with uncertainty and/or decisions in the future.

*When I look at [your] file, and my colleague did as well, we would have started the treatment in the same way. […] But we can speculate together about “what if.” What if at some point, for example, this treatment no longer works, what would be your options by then? There is not just one option, there are multiple, which I am going to discuss with you now*. (female oncologist, female patient).

#### Variations in the Choice of Words/Language to Convey Uncertainty

Finally, throughout all consultations we identified variation in language use or choice of words by oncologists when communicating about uncertainty. These variations particularly included oncologists' level of explicitness and use of personal pronouns. Oncologists would alternate between different degrees of explicitness and pronouns between and even within consultations.

##### Level of Explicitness

We identified strong variation in how explicitly oncologists expressed uncertainty. On one end of the continuum, oncologists could be very explicit in their expression of uncertainty, by directly stating something was unknown. On the other end, very implicit expressions of uncertainty were used, entailing subtle vocabulary, such as *maybe, might* or *hope*, to express uncertainty. Yet, even within the same consultation, an oncologist would sometimes express uncertainty explicitly and sometimes implicitly. Overall, it appeared that oncologists used more explicit expressions when they wanted to emphasize unavoidable uncertainty.

*That [side effects at end of life] varies widely. I mean, anything can happen. It is impossible to predict how things will turn out. You can't really make a meaningful statement. That is of no use to you. I just don't know. We cannot predict how it will go for you. I just don't have that, I just don't know*. (male oncologist, female patient)

In contrast, oncologists appeared to use more implicit language particularly when discussing uncertain aspects of which they wanted patients not to become overly conscious or unnecessarily worried about. For example, the following quote includes several implicit expressions of uncertainty (i.e., *hope, less*):

*You hope that because of that [treatment], the swelling decreases a bit and that you suffer less as a result. And eventually, you hope that the chemotherapy will do its job and that [the tumor] will shrink and that you will experience less pain as a result*. (female oncologist, male patient).

##### Use of Personal Pronouns

Oncologists also varied in using the first person pronoun “I” to express uncertainty and the plural pronoun “we.” Again, this tended to differ both across and within consultations, as oncologists constantly switched between these personal pronouns. It appeared that sometimes more complex and unpredictable information was conveyed using “we” (as in “the medical community”) instead of “I,” possibly acknowledging that the uncertainty is inevitable and not due to personal incompetence.

*As doctors we are unable to predict life expectancies. Especially if a patient is sitting in front of you in a stable condition. If people are in hospital and are very sick, you could say: this will take a few days. That we can do. But everything in between, we cannot say*. (male oncologist, female patient).

## Discussion

This study explored how uncertainty is communicated by medical oncologists during second opinion (SO) consultations. By virtue, SOs entail a high level of uncertainty and we found a wide variety of approaches that oncologists used to communicate uncertain information. These communication approaches entailed the extent to which oncologists specified, downplayed or magnified uncertain information, and the amount of support they offered to patients while discussing uncertainty, as well as the language they used. Such ways of communicating about uncertainty may influence patients' perception of uncertainty, emotional response to it, and/or subsequent behaviors, which warrants further research.

Previous research indicated that communication about uncertainty may have contradictory effects on patients. Some results suggested that physicians' expressions of uncertainty led to enhanced patient satisfaction, whereas other studies found reduced trust and satisfaction (Parascandola et al., 2002; Denberg et al., 2006; Blanch et al., 2009; Mishel et al., 2009; Han et al., 2011; Cousin et al., 2013). Importantly, these studies focused on the presence vs. absence of communicating about uncertainty, and their opposing findings were proposed to result from varying manners of communication (Gordon et al., 2000), but which manners remained unknown. Thus, insight into approaches that clinicians use to discuss uncertainty was still lacking. This study identified seven approaches to discussing uncertain information with patients, which could have profound effects on patients. For example, by explicitly expressing uncertainty and clarifying the reasons for being uncertain, oncologists may facilitate a shared understanding of why uncertainty exists (Blanch et al., 2009). This experience of “shared uncertainty”—where uncertainty resides in the minds of both the physician and the patient—may reassure patients and enhance their trust (Hillen et al., 2017a). In contrast, oncologists may remain implicit and/or omit specific uncertain information in an attempt to protect patients from experiencing strong emotions by not overly emphasizing uncertainty and potentially worrying them (Stortenbeker et al., 2019). However, this could cause patients to be oblivious to the severity of their situation and unable to take well-informed decisions (Politi et al., 2011). In fact, previous research indicated that oncologists often remained vague about patients' prognosis, which may hinder not only patients' understanding of it, but also a proper discussion of treatment goals (Chou et al., 2017). Our analysis showed various approaches, in which oncologists would express uncertainty explicitly, but still provide patients with some guidance in dealing with it. For example, oncologists would sketch different scenarios for future treatment options, sometimes tailored to the patient specifically. This may be particularly helpful in the setting of providing a SO, because many patients are motivated to seek a SO due to a perceived lack of personalized information from their treating oncologist [i.e., the “first opinion” (Goldman et al., 2009)]. By utilizing some of the communication approaches identified in the present analysis, oncologists could ensure that patients are aware of existing and unavoidable uncertainties, while reducing the emotional impact on patients (Brookes-Howell, 2006; Goldman et al., 2009; Santhosh et al., 2019).

Oncologists are tasked with providing a delicate balance between openly informing patients about uncertainty while protecting them against harmful effects caused by uncertainty. This trade-off is different for each patient, and may be strongly affected by their individual coping styles and overall ability to deal with uncertainty (Hillen et al., 2017b). For example, patients with a more active problem-solving style were found to appreciate oncologists who explicitly express uncertainty, whereas patients with an avoidant coping style preferred expressions of non-disclosure (Mori et al., 2019). Thus, providers may try to gauge patients' individual preferences, beliefs, and coping styles regarding uncertainty. They could do so by checking patients' existing knowledge, beliefs and feelings about uncertainty, and by exploring how much (more) patients want to hear (Seely, 2013; Pino and Parry, 2019). This would enable providers to tailor their level of explicitness and detail when conveying uncertain information to individual patients. Importantly, oncologists in our study personalized risk estimated to individual patients, but we did not observe that they checked and adjusted to whether and which amount of (uncertain) information the respective patient may have wanted to receive.

This study identified several ways in which oncologists may have (un)consciously steered patients' perception of uncertainty, and possibly their subsequent emotions and behavior. For example, we found that oncologists sometimes downplayed or magnified uncertain information in an apparent attempt to influence patients' treatment choice, particularly regarding participation in clinical trials. The option of participating in a trial is often a motivator for cancer patients to seek a SO, which may be why we observed this approach regularly in this setting. However, previous research also found oncologists in regular breast cancer care to use similar implicit persuasive behaviors to convince patients of the treatment option they believed was in the patient's best interest (Engelhardt et al., 2016). While oncologists may indeed have patients' best interest in mind when trying to persuade a patient, it could also have harmful effects. For example, downplaying certain treatment side-effects in an effort to steer patients toward a treatment option could bias patients' perceptions, undermine their autonomy, and/or leave them unable to make a well-informed decision between treatment options. Oncologists' opinions can carry a lot of weight and may impede patients in forming their own opinion (Engelhardt et al., 2018). More subtly, even smaller word choices, like using the pronoun “we” instead of “I” may affect patients' perception of uncertainty. The phrase “we don't know,” referring to the whole medical or research community, carries much weight and may lead patients to believe that uncertainty is inevitable, compared to when the oncologist admits his/her own lack of knowledge by using “I” (Juanchich et al., 2017). Oncologists may not deliberately choose these pronouns each time, but patients can take such linguistic markers into consideration when interpreting uncertain information (Juanchich et al., 2017). Oncologists may need to be extra aware of the different effects their words can have on patients, and of the benefits, drawbacks, and ethical dilemmas in relation to steering patients' perception of uncertainty.

This study further identified that oncologists sometimes actively provided emotional support to patients or counterbalanced uncertainty, seemingly in an effort to help patients deal with uncertainty. Considering the extensive length of these SO consultations (i.e., *M* = 41 min), oncologists have ample opportunities and time to use such “supportive” strategies compared to regular consultations, which are often characterized by time constraints. Nevertheless, such strategies were only observed occasionally in the analyzed consultations. It is recommended that physicians provide emotional support during the complete care process (Armstrong, 2018; Simpkin and Armstrong, 2019), and patients reported to desire this commitment and engagement from their providers (Srivastava, 2011). Although SOs usually involve only one or two consultations, oncologists providing SO consultations could still provide support by actively asking patients how the discussed uncertainty affects them, or by explaining the optimal next steps for patients (Santhosh et al., 2019). For example, oncologists in this study directly asked about patients' emotional reaction to uncertainty, normalized such reactions, or discussed different scenarios that might benefit the patient in the future. Two previously conducted observational studies found that both patients and caregivers highly valued it when the physician emphasized which elements of an uncertain situation they *could* control (Cagle et al., 2016; Olson et al., 2018). Thus, discussing different potential scenarios may benefit cancer patients' emotional responses to uncertainty as well. Moreover, we found that oncologists counterbalanced uncertain with certain information, which they appeared to do in an effort to reduce the psychological burden of uncertainty on patients. A similar strategy was also identified in regular oncological consultations, where oncologists were observed to sometimes alternate uncertain news with more reassuring news (Alby et al., 2017). Overall, such strategies to support patients in dealing with uncertainty are encouraged to be used, and may benefit patients and their families directly.

Although this study offers valuable, in-depth insights into how oncologists communicate uncertain information in SO consultations, some limitations need to be considered. First, our analysis explicitly focused on identifying communicative approaches by oncologists, and did not incorporate patients' responses. Future studies may assess the differential effects of different approaches to convey uncertainty on patients. Ideally, such research would use video instead of audio recordings, to also allow capturing patients' non-verbal responses. Thereby, analyses of patients' direct responses should be complemented with self-report data to provide comprehensive insight into patients' emotions and perceptions. A second limitation is that our analysis focused on SOs in medical oncology, which are particularly long consultations that can entail a high degree of uncertainty. Therefore, the variety of approaches to communicating uncertainty may have been particularly rich due to these specific characteristics, but it remains unclear to what extent our findings can be extrapolated to “regular” oncological or medical consultations. Nevertheless, several “supportive” approaches to communicating uncertainty illustrated here could be useful to providers in various setting. Third, our sample size is limited, but included a purposively selected sample of female and male oncologists with high and low PCC scores to increase variability and ecological validity. We also invested extensive time to double-code and discuss all consultations to minimize potential coder bias and maximize the reliability of our identified communication approaches. Fourth, our audio-recordings did not allow for coding non-verbal behavior, whereas this may also play a relevant role in patient-provider communication (Ogden et al., 2002). Finally, we want to highlight that certain intentions of oncologists (e.g., persuasion/steering) were inferred on our behalf as we judged them as apparent in the consultations. These may not always have been the conscious intentions of oncologists and we were unable to verify oncologists' intention. Yet, and irrespective of whether intended or not, patients and families may also pick up on such communication behaviors, which could be experienced positively by some (e.g., appreciating a clear preference) and negatively by others (e.g., feeling one's autonomy compromised). It remains to be tested how such communicative behaviors affect patients' perceptions of the oncologist and consultation.

To conclude, this study contributes to the limited empirical evidence by identifying different approaches to how oncologists communicate uncertain information during SOs. We found variation in the degree of specifying/magnifying uncertainty, offering support in dealing with uncertainty, and language use between and within consultations. These different approaches to communication may affect patients' perception of uncertainty, the emotions provoked by it, and possibly even their behavior. In clinical practice, oncologists need to be conscious of the potential effects of their communication on patients, and use communication approaches purposively and carefully. More research is needed to examine how various ways of communicating about uncertainty affect patients. Such evidence could also facilitate a discussion about the desirability of certain communication strategies. Eventually, practical guidance should be developed for clinicians to optimally inform patients about uncertain issues and support them in dealing with it.

## Data Availability Statement

The data analyzed in this study are subject to the following licenses/restrictions: Anonymized parts of the dataset may be viewed upon request. Requests to access these datasets should be directed to Marij A. Hillen, m.a.hillen@amsterdamumc.nl.

## Ethics Statement

The studies involving human participants were reviewed and approved by Medical Ethics Committee Academic Medical Center Amsterdam, The Netherlands (NL63087.018.17). The patients/participants provided their written informed consent to participate in this study.

## Author Contributions

JLS was involved in conceptualization, methodology, data preparation, conducting the analysis, project administration, and writing (original draft, review, and editing). VL was involved in conceptualization, methodology, conducting and supervising the analysis, project administration, and writing (original draft, review, and editing). JMS was involved in conceptualization, providing resources, and writing (review and editing). AS was involved in conceptualization and writing (review and editing). ES was involved in conceptualization, methodology, supervising the analysis, and writing (review and editing). MH was involved in funding acquisition, conceptualization, methodology, conducting and supervising the analysis, data curation, project administration, supervision, and writing (original draft, review, and editing). All authors contributed to the article and approved the submitted version.

## Conflict of Interest

The authors declare that the research was conducted in the absence of any commercial or financial relationships that could be construed as a potential conflict of interest.
