# Supplementary material for: Oncologists' Communication About Uncertain Information in Second Opinion Consultations: A Focused Qualitative Analysis
Source: Front Psychol. 2021 May 31;12:635422. doi: 10.3389/fpsyg.2021.635422 (PMC8201772; doi:10.3389/fpsyg.2021.635422)
Supplement: Supplementary file 1 [file Table_1.DOCX]

Table 1: Overview of identified strategies for communicating about uncertainty

|  | **Strategy** | **Definition/scope** |
| --- | --- | --- |
| **1** | Specifying the degree  of uncertainty | Using generic/vague terms (e.g., ‘much’) or more specific estimates (e.g., ‘20%, that is 1 in 5 patients’) to qualify or quantify the degree of uncertainty. |
| **2** | Explaining reasons of uncertainty | Clarifying underlying reasons/providing explanations for why a situation or particular information is uncertain, e.g., by acknowledging the limits of medical science. |
| **3** | Providing personalized estimates of uncertainty | Tailoring uncertain information to a specific patient’s characteristics and/or situation, to provide individualized estimates. |
| **4** | Downplaying or magnifying uncertainty | Minimizing or emphasizing uncertainty, seemingly to influence patients’ perceptions, emotions and/or behavior |
| **5** | Reducing or counterbalancing uncertainty | Trying to directly reduce uncertainty for patients, e.g., by offering reassuring information, or counterbalancing uncertainty by emphasizing certain aspects. |
| **6** | Providing support to facilitate patients in coping with uncertainty | Offering emotional support to help patients cope with uncertainty and/or normalizing worries. |
| **7** | Choice of words/ language to convey uncertainty | Utilizing different language or words to express uncertainty:  - implicit *vs.* explicit language: ‘maybe’ *vs.* ‘I don’t know’  - first *vs.* third person pronoun: ‘I’m not sure’ *vs.* ‘we [as doctors] are not sure’ |
